# Marine population-genetic inferences reveal stronger oceanic structure in protists than Archaeplastida (plants) and Metazoa (animals)

**DOI:** 10.1126/sciadv.adz7158

**Published:** 2026-06-10

**Authors:** Rubén González-Miguéns, Alex Gàlvez-Morante, Guifré Torruella, Cédric Berney, Elena Casacuberta, Iñaki Ruiz-Trillo

**Affiliations:** ^1^ Institut de Biologia Evolutiva (CSIC-Universitat Pompeu Fabra), 08003 Barcelona, Spain.; ^2^ ICREA, 08010 Barcelona, Spain.

## Abstract

While the biogeographic patterns of animals and plants are well defined, the distribution of the microbial eukaryotic world remains contentious. Classically, protists were assumed to be cosmopolitan, dispersing everywhere with little geographic constraint. We tested this hypothesis by compiling 88 marine cytochrome oxidase subunit I metabarcoding studies to perform a global population-genetic analysis. We uncovered a fundamental biogeographic divide: most animal (Metazoa) and plant (Archaeplastida) phyla have haplotype diversity that is strictly concentrated within specific ocean basins, while most protist phyla share identical haplotypes across multiple, distant oceans. The combination of large geographic ranges with moderate among-ocean genetic differentiation in protists is inconsistent with strict historical cosmopolitanism and is compatible with high connectivity and range expansion dynamics, including, but not limited to, recent colonization events. Our findings provide a unified population-genetic perspective on marine eukaryotic biogeography and refine current views of microbial dispersal at ocean-basin scales.

## Teaser

Global metabarcoding reveals contrasting oceanic population-genetic structure between protists and multicellular eukaryotes.

## INTRODUCTION

Unicellular eukaryotes (protists) have classically been viewed as fundamentally different from multicellular plants and animals with regard to their biogeographic patterns (*1*). Early theories postulated that protists have cosmopolitan distributions and virtually unlimited dispersal capacity, giving rise to the famous concept “everything is everywhere, but the environment selects” (*2*). This perspective, however, was largely grounded in morphological observations, which, due to their limited resolution, often overlooked cryptic diversity. Subsequent molecular work has shown that protist populations can nonetheless display clear genetic differentiation across regional and even ocean-basin scales (*3*–*5*) and that spatial structuring frequently plays a stronger role than local environmental variation in shaping diversity (*6*). Moreover, both widely distributed and geographically restricted protist lineages have now been documented (*7*, *8*). Together, these findings suggest that the “everything is everywhere” concept is best treated as a null expectation rather than a universal rule.

Advances in large-scale molecular techniques, such as metabarcoding, now allow much more precise assessments of protist biogeography (*9*) and facilitate comparison of patterns across ecosystems. These molecular tools provide an opportunity to explicitly test long-standing assumptions about geographic connectivity in marine environments. Marine systems are often assumed to promote high connectivity due to the absence of major physical barriers (*10*). If so, then marine protists, characterized by high dispersal rates, would be expected to show weak spatial genetic structure relative to multicellular eukaryotes. However, several molecular studies report substantial biogeographic differentiation in marine protists, including restricted distributions that sometimes parallel those observed in macroorganisms (*4*, *8*, *11*, *12*). Nevertheless, these investigations have predominantly focused on presence-absence matrices, β-diversity patterns, and relative abundances inferred from read depth. A critical knowledge gap persists regarding how population-level genetic diversity shapes the distributions of protists, as it provides historical context for present-day biogeographic patterns (*13*). Even when species appear broadly distributed, their populations may differ genetically across regions, revealing the processes that structure diversity at large scales. Understanding this structuring is essential to determine whether the biogeographic principles described for multicellular animals (Metazoa) and plants (Archaeplastida) are truly universal across eukaryotes or whether protists present specific biogeographic patterns.

To characterize the protists’ biogeographical patterns, metabarcoding represents a promising approach (*14*), as the large amount of molecular and geographic data from different organisms enables the integration of geographical and population genetic theories within a unified biogeographic framework (*15*–*17*). However, its effectiveness depends on meeting certain conditions: (i) the use of molecular markers with intraspecific resolution [for example, the mitochondrial cytochrome oxidase subunit I gene, COI, which has proven invaluable in animal biogeography (*18*)] and some protists (*9*, *19*–*21*); (ii) the availability of a comprehensive global database for these markers that includes data from both protists and multicellular eukaryotes (*22*); (iii) the use of standardized taxonomic units that mitigate the inherent biases of metabarcoding (*23*); and (iv) the integration of population genetics methods and theory into metabarcoding analyses.

Here, we address these limitations by compiling 88 marine COI metabarcoding studies and integrating them into a unified eukaryotic, cross-phyla biogeographic framework. Using population-genetic metrics at a global scale, we compare the spatial molecular structure of protists with that of animals (Metazoa) and plants (Archaeplastida) to evaluate whether protists exhibit fundamentally distinct spatial genetic organization and whether their patterns are consistent with strict historical cosmopolitanism or alternative demographic scenarios.

## RESULTS

### Construction of the eKOI database with informative operational taxonomic units to test biogeographic patterns

**Fig. 1.**
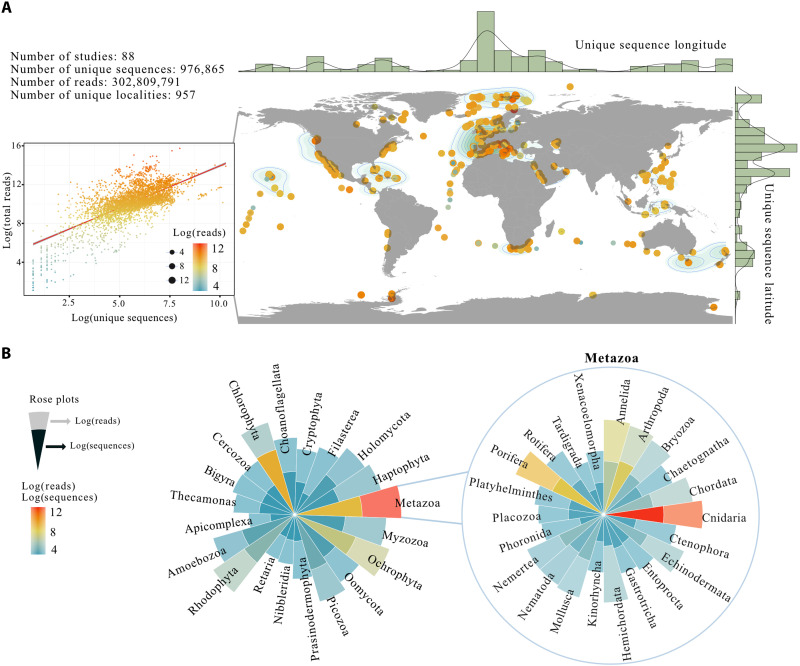
eKOI metabarcoding database. (**A**) Map showing the locations of samples included in the database. The size and color of the points represent the log(total reads) for each sample. To the left of the map, a scatter plot displays the relationship between log(ASVs) and log(reads) for the sampled localities, colored according to log(reads). (**B**) Rose plots representing the proportion of taxonomic annotated ASV as log(ASV) on the inner layer and log(reads) on the outer layer in the different eukaryotic phyla used in the analysis.

To investigate global eukaryotic biogeographic patterns at the population level, a critical first step is the creation of a comprehensive metabarcoding database amenable to population-genetic analyses. So, we here compiled data from 88 independent metabarcoding studies, including both environmental DNA (eDNA) and bulk community samples from plankton and sediment, and covering coastal and open-ocean environments, into the eKOI metabarcoding database; comprising 976,865 amplicon sequence variants (ASVs) with no abundance filter (fig. S1) and 302,809,791 reads from 4102 samples collected at 957 distinct sites that span all oceans (Fig. 1A). To ensure comparability, we selected only studies that used widely used universal eukaryotic COI primers (*24*–*27*), which have been shown to perform well in protists, recovering a broad diversity of phyla (*22*). We then taxonomically annotated them at phylum level, comprising all eukaryotes including animals and plants, and clustered them into 51,558 operational taxonomic units (OTUs), using a 3% divergence cutoff (see Materials and Methods). All analyses were conducted at the OTU level, incorporating the full set of ASVs contained within each OTU.

To ensure consistency across the major eukaryotic lineages, we classified taxa following current phylogenomic frameworks. Accordingly, we included within Metazoa all monophyletic animal phyla and within Archaeplastida all lineages recovered as part of this clade in recent phylogenomic analyses. Archaeplastida therefore includes not only multicellular plants but also unicellular green algae, red algae, and Picozoa, the latter being placed within Archaeplastida in recent phylogenomic studies (*28*). In contrast, the group commonly referred to as “protists” does not represent a monophyletic lineage but rather a paraphyletic assemblage of diverse eukaryotic phyla.

**Fig. 2.**
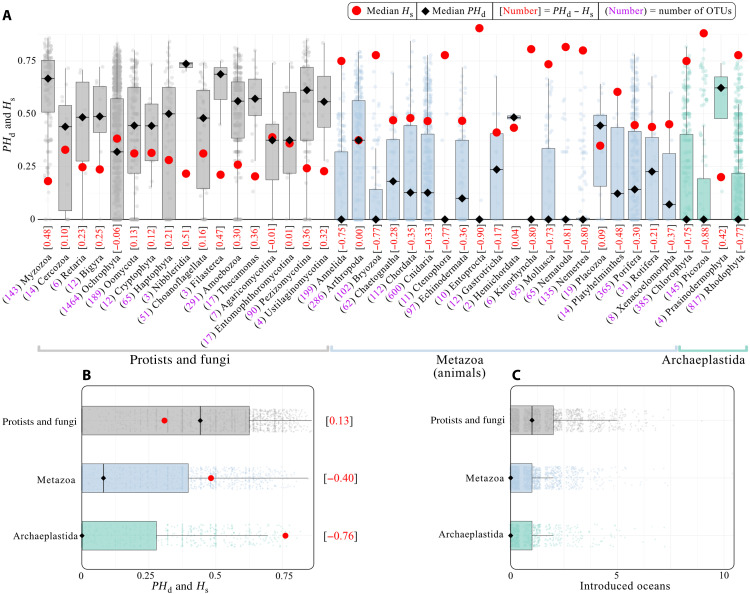
Population structure between eukaryotic phyla. (**A**) Box plots representing the *PH*_d_ values for each OTU between phyla, where values close to 0 indicate low structuring in terms of intra-OTU haplotype diversity. Red circles with crosses represent the median *H*_s_ values for each phylum. The number near each phylum name represents the number of OTU recovered after filtering. The numbers at the top indicate the difference between *PH*_d_ and *H*_s_. (**B**) *PH*_d_ values grouped by different phyla. (**C**) Number of recently introduced oceans per OTU grouped by different phyla.

To ensure robust population-genetic analyses and minimize potential artifacts, we stringently filtered the data to retain only informative OTUs, defined as those (i) containing at least four sequences from a minimum of two distinct locations and (ii) containing at least one pair of sequences that differ by ≥1% (uncorrected *P* distance). This two-step filtering removed OTUs with very few sequences or negligible variation, thereby reducing spurious OTUs from methodological biases and improving the reliability of biogeographic patterns (see Materials and Methods). The final dataset comprise 5970 informative OTUs, including 276,303 ASVs and 101,898,078 reads distributed across 41 phyla (Figs. 1B and 2A). Crucially, post hoc pairwise comparisons of nucleotide diversity (π) and Tajima’s *D* among phyla revealed that only 0.001 and 6.9% of the comparisons, respectively, were significant (Dunn’s test with Bonferroni correction; see Materials and Methods, fig. S2, and Supplementary Data S4), confirming broadly comparable levels of nucleotide diversity (π) and Tajima’s *D* across phyla, minimizing the risk that systematic differences in baseline genetic diversity could bias our biogeographic inferences. Consequently, this constructed eKOI metabarcoding resource provides standardized taxonomic units, thereby establishing a solid foundation for rigorous, population-level investigations of marine biogeographic hypotheses.

### Protists have larger geographic ranges than animals and plants

A long-standing debate in eukaryote biogeography concerns whether protists have larger geographic ranges than macro-organisms (*1*). Testing this hypothesis has been hindered by the sheer volume of molecular and geographic species-level data required at a global scale (*29*). Our eKOI database allows for a direct assessment of this question. To quantify the geographic range of each OTU, we used two standard distance-based proxies: (i) the mean pairwise geographic distance among all sampling localities where the OTU was detected, which reflects its overall spatial spread, and (ii) the maximum distance between any two localities, representing the linear extent of its range. We then compared these OTU-level range metrics across phyla. We found that OTUs assigned to Metazoa and Archaeplastida typically show mean interlocality distances around 1000 km, whereas OTUs from most protist phyla average over 5000 km, with some exceeding 10,000 km (fig. S3). Post hoc pairwise comparisons across phyla, based on the median range metrics of the OTUs within each phylum, showed that these differences are significant for both metrics (19.02% for mean pairwise geographic distance and 18.17% for maximum interlocality distance; Dunn’s test with Bonferroni correction; see Materials and Methods and Supplementary Data S4). This demonstrates that protists, at the OTU level defined by COI, present considerably larger geographic ranges than plants and animals.

### Molecular and geographic expansions in protists

To understand the spatial processes underlying the broad geographic ranges observed in protist OTUs across phyla, additional molecular data are essential, since they enable the detection of within-OTU genetic patterns linked to geographic range (*16*). Therefore, we compared genetic versus geographic distances within each informative OTU to test whether the observed spatial patterns were nonrandom and consistent across different phyla. Our analyses using both linear and nonlinear regressions revealed that most OTUs displayed significant nonrandom spatial patterns. Specifically, when compared to null models (see Materials and Methods), 81.5% of OTUs in the linear models and 92.6% in the nonlinear models had *R*^2^ values that were statistically significant (*P* < 0.05, obtained through simulations; see Materials and Methods and Supplementary Data). Furthermore, linear and nonlinear models (fig. S4), as well as Mantel tests (fig. S5), consistently showed positive relationships between genetic and geographic distances across all eukaryotic phyla examined, consistent with isolation by distance (IBD). That is, genetic divergence tended to increase with greater geographic separation.

Having established that most informative OTUs exhibit nonrandom spatial pattern, IBD-consistent structure (figs. S4 and S5), we next quantified the strength of this genetic and geographic relationship. We used the slope of the linear model and the parameter *a* of the exponential model as effect size summaries of how rapidly genetic divergence increases with distance. These effect sizes were broadly uniform across phyla, with significant between-phyla differences being rare (5.7 and 3.6% of pairwise comparisons, respectively; Dunn’s test with Bonferroni correction; see Materials and Methods). Moreover, when comparing across OTUs, the geographic extent of an OTU (mean intersample distance) was only very weakly correlated with its genetic divergence, indicating a limited association between range size and the strength of IBD. Thus, although most OTUs display statistically detectable IBD, this relationship is generally weak and does not systematically increase with geographic extent. In practical terms, genetic divergence tends to increase with geographic separation, but geographic range accounts for only a small fraction of the observed variation in IBD strength. Larger geographic ranges do not translate into stronger spatial genetic structuring, and OTUs with vast distributions often show levels of genetic differentiation comparable to those with much smaller extents. Consequently, geographic distance alone appears to have limited association with patterns of genetic differentiation across phyla, even over ocean-basin scales.

### Metazoa exhibit higher richness of haplotypes among eukaryotes

To follow up on those rapid geographic expansions, we then inquired how and when current OTU distributions were established. To do this, we performed explicit population-genetic analyses based on haplotypes, aiming to determine how molecular diversity is structured within each informative OTU. Before conducting these analyses, we evaluated whether haplotype-based metrics could be biased by sequencing depth within each OTU. Haplotype richness showed only a weak correlation with sequencing depth, suggesting that variation in read depth was not strongly associated with estimates of haplotype diversity. This limited association is consistent with the use of this metric for comparative population-genetic analyses in metabarcoding datasets, with reduced sensitivity to differences in sampling depth. Using the haplotypes identified within each OTU, we constructed haplotype networks for each informative OTU, without imposing a strictly bifurcating tree topology (*30*). This network-based approach allowed us to infer intra-OTU genealogies while avoiding the assumption of purely bifurcating relationships inherent to traditional phylogenies, which may not accurately capture genetic structure at the population level (*31*).

As a first step, we characterized haplotype diversity distributions across major phyla using three complementary metrics, each calculated for each OTUs independently: haplotype diversity (*H*_d_) (*32*), branch diversity (*B*_d_), and a combined haplotype-branch diversity index (*H*_bd_) (*33*). Haplotype diversity (*H*_d_) measures the probability that two randomly sampled sequences represent different haplotypes and is widely used as an indicator of genetic variation (*32*, *33*). Higher *H*_d_ values indicate greater richness of haplotypes and thus more genetic diversity within OTUs or populations, whereas lower values suggest reduced diversity and potential demographic constraints. These metrics were calculated on the basis of the number of ASVs (haplotypes), the total read abundance per haplotype, and log-transformed read counts. *B*_d_ and *H*_bd_ values showed relative consistency across metazoan phyla, with few significant interphylum differences (fig. S6). In contrast, *H*_d_ exhibited greater variation across phyla, primarily driven by comparisons involving Metazoa, which consistently displayed near-maximal *H*_d_ values. This indicates a significantly higher richness of haplotypes and, consequently, greater intra-OTU genetic diversity within metazoan OTUs compared to those of other eukaryotic phyla.

### Genetic structuring among oceanic regions in marine eukaryotes

Following the characterization of within-phylum haplotype diversity, we next assessed genetic structuring across oceanic regions. To do this, we performed explicit population-genetic analyses to determine how molecular diversity is partitioned within each informative OTU. In this framework, each oceanic region was treated as an independent population. For each OTU, we quantified haplotype frequencies and their distribution across these oceanic regions (=populations). We quantified population-level differentiation using four standard metrics of population genetic structure: total genetic diversity (*H*_t_), within-ocean genetic diversity (*H*_s_), the fixation index (*F*_st_) (a measure of among-ocean differentiation) (*34*), and the effective number of migrants (*N*_m_) (an indirect estimate of gene flow) (*35*). These metrics revealed significant differences among phyla in their degree of genetic structuring, with 20% of interphyla comparisons for *H*_t_, 9.7% in *H*_s_, 21.9% in *F*_st_, and 9.3% in *N*_m_ recovering significant results in post hoc pairwise tests (Dunn’s test with Bonferroni correction; see Materials and Methods and fig. S7). The most pronounced disparities were observed in comparisons involving OTUs within Metazoa and Archaeplastida versus OTUs in other phyla. Specifically, Metazoa and Archaeplastida showed higher *H*_s_ values, largely reflecting that many OTUs occur in only one or very few oceans, which inflates within-ocean genetic diversity (*H*_s_). Because *F*_st_ is calculated independently for each OTU, OTUs restricted to a single ocean necessarily yield *H*_s_ ≈ *H*_t_ and therefore *F*_st_ ≈ 0, regardless of their broader phylogeographic structure. Consequently, despite the presence of numerous ocean-specific haplotypes, *F*_st_ values for the phyla of Metazoa and Archaeplastida remained consistently low (often approaching 0 and never exceeding 0.5). In contrast, protist phyla exhibit broader distribution of OTUs across multiple oceans, lower *H*_s_, and consistently high *F*_st_ values (≥0.5), indicating limited sharing of dominant haplotypes across basins and stronger among-ocean genetic structuring (fig. S7).

To address the limitations of *H*_s_, in capturing haplotype distribution across oceans, we developed the population-level haplotype diversity (*PH*_d_) metric, which integrates intraoceanic diversity and the evenness of haplotype distribution across oceans (see Materials and Methods). *PH*_d_ penalizes uneven distributions, and the difference between *PH*_d_ and *H*_s_(*PH*_d_ − *H*_s_) indicates the degree to which an OTU’s genetic diversity is structured among oceans. Positive values (*PH*_d_ > *H*_s_) suggest that haplotypic diversity is partitioned across oceans, consistent with genetic structuring, whereas negative values (*PH*_d_ < *H*_s_) indicate that one or a few oceans dominate the haplotypic diversity. *PH*_d_ analysis revealed significant interphylum differences, 20.6% of comparisons, largely mirroring *H*_s_ and *F*_st_ trends. Metazoa and Archaeplastida (including unicellular red and green lineages) showed near-zero *PH*_d_ values and negative *PH*_d_ − *H*_s_ differences, suggesting concentrated haplotypic diversity in limited oceanic regions (Fig. 2, A and B). However, some metazoan groups, such as Arthropoda (*PH*_d_ − *H*_s_ = 0), Gastrotricha (−0.17), Placozoa (0.09), and Rotifera (−0.21), exhibited more even haplotype distributions. Protist phyla generally showed slightly higher *PH*_d_ values and *PH*_d_ − *H*_s_ differences close to zero, indicating moderate population genetic structuration. However, caution is required when interpreting large and heterogeneous phyla such as Ochrophyta, within stramenopiles, which encompass lineages with very different evolutionary histories, for example, multicellular brown algae (Phaeophyceae) versus closely related unicellular taxa (e.g., diatoms). When considering all OTUs assigned to Ochrophyta, the *PH*_d_ − *H*_s_ difference is close to zero (−0.06; Fig. 2A). However, when separating them, the predominantly unicellular lineages yield values close to zero (−0.055), whereas Phaeophyceae OTUs show more negative values (−0.12), similar to those observed in multicellular Metazoa and Archaeplastida.

Overall, *PH*_d_ and *H*_s_ values revealed patterns of broad geographic ranges coupled with limited genetic divergence. Such patterns can arise from several, not mutually exclusive, processes, including high dispersal capacity, low geographic barriers, or ecological isolation that preserves similar genetic structure across habitats. Comparable patterns are also frequently observed in species undergoing recent introductions or rapid geographic expansions, where geographic range increases faster than genetic divergence can accumulate (*36*, *37*). While these observations may be consistent with recent colonization in some cases, they can equally reflect other demographic or ecological processes and should therefore be interpreted with caution.

### A potential hypothesis: Recent introductions shaped the current biogeography of protists

The *PH*_d_, *H*_s_, and IBD patterns together reveal that many protist OTUs combine broad geographic ranges with uneven haplotype partitioning across oceans. These patterns can arise under multiple, nonexclusive demographic scenarios, including high dispersal, episodic range expansion, or recent colonization events in which geographic spread outpaces the accumulation of mitochondrial divergence. Here, we explore the latter as a working hypothesis, while acknowledging that alternative explanations may generate similar biogeographic signatures.

We therefore used the *PH*_d_ metric to test whether the basin harboring the majority of an OTU’s haplotypic diversity constitutes a plausible source (“dominant ocean”), whereas basins with comparatively lower haplotypic diversity represent putative recipient regions (“introduced oceans”). From a population-genetic perspective, these asymmetries in haplotypic diversity can be informative: Donor regions (dominant ocean) are expected to retain higher diversity due to longer evolutionary history and larger effective population sizes, while recently colonized recipient regions (introduced oceans) may display reduced diversity as a result of founder effects. These discrepancies can therefore provide indirect evidence of recent colonization or introduction events. However, caution is required when characterizing species as native or introduced, as the evolutionary history of some species remains unclear with the current data (i.e., cryptogenic species) (*38*). We emphasize, nonetheless, that this interpretation remains a hypothesis and must be tested on a case-by-case basis for each OTU.

Consistent with this expectation, across most metazoan phyla (Annelida, Bryozoa, Chaetognatha, Chordata, Cnidaria, Ctenophora, Echinodermata, Entoprocta, Hemichordata, Kinorhyncha, Mollusca, Nematoda, Nemertea, Placozoa, and Platyhelminthes), as well as phyla within Archaeplastida (Chlorophyta, Picozoa, and Rhodophyta) and Ochrophyta, we observed minimal evidence of multiocean distributions (Fig. 2C and fig. S8). In contrast, other metazoan phyla (Arthropoda, Gastrotricha, Porifera, Rotifera, and Xenacoelomorpha) often showed broader distributions, averaging around one introduced ocean per OTU (fig. S8). Similarly, most protist phyla frequently displayed one or more introduced oceans per OTU, typically with low haplotypic diversity. These patterns may reflect relatively recent interocean colonization events, where geographic range expansion has occurred faster than substantial COI divergence could accumulate. Throughout this section, “recent” refers to short evolutionary timescales relative to mitochondrial divergence at COI, potentially spanning decades to a few thousand years. We do not imply a specific temporal window, nor do we assume exclusively anthropogenic drivers.

As a validation step, we asked whether our framework can recover well-documented introduction events in OTUs. We focused on OTUs taxonomically assigned to Chordata, where introduction histories are comparatively well described (*39*). Our analysis successfully highlighted several Chordata OTUs corresponding to species known for human-mediated introductions or widespread dispersal, including the fishes *Acipenser gueldenstaedtii*, *Salmo trutta*, and *Siganus rivulatus*, as well as the tunicates *Botryllus schlosseri* and *Styela plicata*. These cases support the ability of our approach in identifying recent introductions from metabarcoding data. Beyond individual OTUs, the inferred geographic connectivity across oceans differed among groups: In Metazoa and Archaeplastida, most inferred source-sink connections, based on haplotypic networks (see Materials and Methods), occurred between geographically adjacent oceans and within the same hemisphere, suggesting predominantly regional spread (fig. S9). In contrast, protist phyla OTUs often showed long-distance dispersal between nonadjacent oceans and across different hemispheres, implying that recent introductions of these microorganisms frequently span distant oceanic regions, far exceeding the regional spread seen in larger eukaryotes (*10*).

**Fig. 3.**
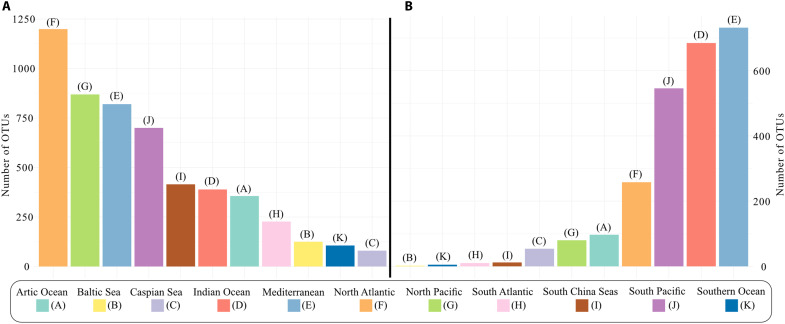
Number of recently introduced and dominant informative OTUs per ocean. (**A**) Box plots showing the number of informative OTUs considered recently introduced in each ocean and (**B**) the number of informative OTUs per ocean identified as their dominant.

Last, by aggregating dominant and introduced ocean assignments per OTU across all phyla, we uncovered broad biogeographic trends that align with known global patterns of species introductions. The highest numbers of introduced informative OTUs were detected in Northern Hemisphere oceans, particularly the Atlantic and North Pacific (Fig. 3), consistent with these regions’ historically high rates of species introductions (*39*–*41*). We note, however, that this pattern may in part reflect sampling biases, as genomic surveys have been more extensively conducted in the Northern Hemisphere. Nonetheless, the fact that OTUs in these oceans were disproportionately classified as introduced rather than as donor lineages, together with extensive prior evidence of high introduction rates in these basins, suggests that the pattern is unlikely to be explained by sampling effort alone. In contrast, the main source regions (dominant oceans) across taxa were the Mediterranean Sea and Indian Ocean, both noted for their high levels of endemic diversity (*42*, *43*). In the case of the Mediterranean, however, many OTUs were also characterized as “introduced,” indicating that this basin can function simultaneously as a major recipient of nonindigenous lineages and as a secondary donor hub for onward dispersal. Overall, these findings underscore that potential recent colonization may have left a distinct imprint on global marine genetic diversity, with identifiable source areas and introduction hotspots explaining the distribution patterns we observe.

## DISCUSSION

Our global COI analysis, leveraging informative OTUs and the innovative *PH*_d_ metric for assessing population-level genetic diversity, reveals fundamental distinctions in the contemporary biogeography of marine eukaryotes. Metazoa (animals) and Archaeplastida generally exhibit significantly restricted ranges and weaker ocean-basin genetic structure compared to protist phyla, underscoring divergent molecular population dynamics across major lineages.

Dispersal plays a fundamental role in shaping molecular population dynamics, community composition, and even speciation. Restrictions to dispersal, whether due to ecological barriers, selective pressures, or life history traits, can lead to geographic constraints where intraspecific diversity accumulates over time. While these patterns are typically observed in marine regions with limited connectivity structured by predominant ocean currents, our results point to a high proportion of potential recent population introductions, particularly among marine protist phyla. These events leave a distinct molecular signature in the structuring of populations across different oceans. Shifts in genetic and biogeographic structure may also reflect contemporary oceanic challenges, including rising temperatures and acidification. However, one of the most disruptive anthropogenic forces affecting marine dispersal is human-mediated dispersion, such as global shipping (*44*), a well-known vector for metazoan introductions (*45*) and recently in protists (*46*). Our results extend this pattern by indicating that protists and small-bodied metazoans (Arthropoda, Rotifera, Gastrotricha, and Placozoa) show heightened susceptibility to long-distance dispersal, as evidenced by elevated *PH*_d_ values compared to other metazoan phyla. The comparatively lower *PH*_d_ observed in these small-bodied metazoans relative to protists may be explained by the latter’s rapid generation times (*47*) and the possibility of multiple introduction events (*48*), both of which could facilitate genetic homogenization in newly colonized oceans and reduce founder effect. Conversely, restricted distributions and weak structure in some small and unicellular Archaeplastida could suggest lower transoceanic transport survival, potentially due to light and resource limitations. Consistent with this explanation, studies of ballast water frequently recover resilient photosynthetic taxa such as Bacillariophyta and dinoflagellates, which are capable of producing resistant forms (*49*), whereas groups such as Chlorophyta appear underrepresented (*50*, *51*). This suggests that ballast water may be a less effective vector for long-distance dispersal in certain Archaeplastida lineages; however, these inferences remain speculative, and alternative ecological or evolutionary processes may produce comparable spatial patterns.

Similarly, several limitations constrain our study and should be considered when interpreting these patterns. First, our analyses describe geographic templates of population structure but do not incorporate environmental drivers; variables such as temperature, salinity, nutrients, or seasonal dynamics (e.g., community shifts due to migration or bloom cycles) may also shape genetic patterns. Second, we relied on a single mitochondrial marker (COI). Although widely used, mitochondrial loci have intrinsic limitations that can bias our interpretations (*52*, *53*): amplification success, genetic variability, and taxonomic resolution vary among clades (*54*, *55*). Third, sampling effort is uneven across oceans, which may bias the detection of introduced or donor regions. Fourth, while we used great-circle distances as a conservative proxy for geographical distances, our IBD results are robust: Positive genetic-geographic associations should persist under any monotonic measure that respects land-ocean barriers. Nonetheless, least-cost paths and oceanographic travel-time models would refine effect sizes within phyla. Last, while our analyses were conducted at the OTU level, our inferences were aggregated by phylum; large groups such as Ochrophyta or Chlorophyta encompass both unicellular and multicellular lineages that may display distinct biogeographic patterns. Future work incorporating additional markers and standardized environmental data will be essential to address these limitations.

Ultimately, our global study establishes a unified framework for interpreting broad biogeographic and population genetic patterns across diverse eukaryotic phyla. The congruent patterns of marine geographic distribution and genetic structure within each group are consistent with shared historical processes, such as recent introductions, shaping their contemporary ranges. These findings refine the traditional view of protists as historically ubiquitously dispersed entities (*1*) at the population genetic level and reveal potentially universal principles governing biogeographic dynamics and community assembly that transcend organismal size and complexity. This framework establishes a quantitative baseline for future studies incorporating additional markers, environmental variables, and temporal data to disentangle the demographic and ecological processes shaping marine microbial biogeography.

## MATERIALS AND METHODS

### eKOI metabarcoding database

To generate the eKOI metabarcoding database, we selected 88 marine metabarcoding studies, with publicly accessible raw sequencing data, that had been generated on an Illumina paired-end platform using the COI molecular marker and used the following primers: HCO (TAAACTTCAGGGTGACCAAAAAATCA) (*27*), LCO (GGTCAACAAATCATAAAGATATTGG) (*27*), jgHCO2198 (TAIACYTCIGGRTGICCRAARAAYCA) (*25*), mlCOIintF (GGWACWGGWTGAACWGTWTAYCCYCC) (*26*), and mlCOIintF-XT (GGWACWRGWTGRACWITITAYCCYCC) (*24*). After obtaining the raw data for each study, we processed each dataset separately according to the protocol outlined in (*22*). As a first step, primer trimming and demultiplexing were performed with Cutadapt (version 2.8) (*56*), when necessary. Next, the resulting reads for each sample in each metabarcoding study were processed independently using the DADA2 R package (*57*), with minor modifications applied depending on the specific metabarcoding study. Chimeric sequences were subsequently removed using the DADA2 *removeBimeraDenovo* function. After inferring the ASVs, we stored them in a FASTA file using the following header format: >sequence_ID merged_sample = {“LOCALITY_ID1”: reads, “LOCALITY_ID2”: reads, ...}. A separate metadata file contained information associated with each locality ID (see metadata.csv in https://doi.org/10.6084/m9.figshare.29144645.v1). For each sample in the metadata, we then assigned the corresponding ocean or sea. To achieve this, we downloaded shapefile datasets from the “Global Oceans and Seas” database (version 1, accessed 11 May 2024) and “Natural Earth” (accessed 11 May 2024). Using each locality’s latitude and longitude coordinates, we determined and recorded the respective ocean or sea for that sample. For subsequent biogeographic analyses, we used the broad ocean regions defined in the Global Oceans and Seas database, which groups the world into 11 major seas and oceans. This approach was chosen to avoid excessive subdivision of oceanic regions.

All FASTA files generated by each metabarcoding study, in the above format, were then merged into a single file (eKOI_metabarcoding_database.fasta in https://doi.org/10.6084/m9.figshare.29144645.v1) using a custom Python script (1_fastas_combine.py). This script uses the Biopython library (*58*) to identify identical ASVs across studies and combine their locality information. Each ASV was assigned an identifier in the format eKOIX (where X is a distinct number for each ASV). Once the final merged FASTA file was assembled, we checked it for chimeric sequences using the VSEARCH *uchime_denovo* command (version 2.14.1) (*59*).

Taxonomic assignment of the ASVs was performed using the eKOI taxonomy database (*22*). For this purpose, we used the script (5_taxonomic_assignation.py) develop in that study. This script created a separate folder for each FASTA file in the working directory. Within each folder, an Excel file was generated containing the taxonomic assignment information for each ASV (Supplementary Data S1 and https://doi.org/10.6084/m9.figshare.29144645.v1), obtained via the VSEARCH *usearch_global* command. ASVs with less than 84% similarity to any reference sequence were not considered further. Last, we generated separate FASTA files for each taxonomic level of interest; in this case, we chose the phylum level. The resulting ASV sequences for each phylum can be downloaded from the “Phyla_data” folder in Supplementary Data as eKOI_database.fasta file per phylum.

### Sequence data processing and analysis

#### OTU clustering

Once FASTA files were generated for each phylum, they were analyzed independently. First, all metadata for each ASV was extracted using the script 2_generate_abundances_unique.py. Biopython was used to extract each ASV’s ID and match it with the metadata file, producing a Dataframe containing the sequence ID, geographic coordinates, reads, and ocean for each record (abundances_unique.csv in https://doi.org/10.6084/m9.figshare.29144645.v1 per phylum). Because some ASVs were found in multiple localities, duplicate entries were created for each locality where an ASV occurred by appending a suffix “_dupN” (where N is the duplicate number) to the ASV’s ID, yielding a distinct identifier for each locality occurrence of that ASV. To avoid biases from multiple sequencing replicates per locality (common in metabarcoding studies), only one ASV entry per locality was retained (duplicate entries with identical ASV ID and coordinates were removed). After this filtering, a comprehensive FASTA file was compiled, including all ASV sequences (with each locality-specific duplicate as a separate entry). This file was aligned using MAFFT version 7.490 (*60*), with optimized parameters (--auto, --ep, --op, --maxiterate, --large) (aligned_sequences_mafft.fasta in https://doi.org/10.6084/m9.figshare.29144645.v1). OTUs were then delineated from the alignment using the script 3_generate_OTU.py. In particular, VSEARCH clustering at 97% identity was used to group sequences into OTUs. We set the clustering threshold at 97% identity (i.e., a 3% divergence cutoff) because COI barcoding studies of Amoebozoa (*61*), Cercozoa (*21*), and diverse animal taxa report similar values (*18*). Although each lineage can display its own diversification rate, published barcoding gaps generally fall between 2 and 3%, so we applied a uniform 3% threshold across all phyla. Representative ASV (centroids) for each OTU were saved, and a mapping of each ASV to its OTU was generated as a .uc file (otus.uc in https://doi.org/10.6084/m9.figshare.29144645.v1 per phylum). The .uc file was subsequently processed to produce a tabular mapping file (otus_mapping.txt in Supplementary Data per phylum) that records the assignment of each ASV ID to its corresponding OTU.

#### Molecular and geographic distance matrix construction and OTU filtering

After obtaining the OTUs, the relationship between genetic and geographical distances among ASVs was analyzed using the R script 4_1_generate_molecular_and_geographic_distances.R. We used the Biostrings R package to convert the phylum-level ASV alignments into DNAbin R objects, suitable for distance calculations. Genetic distance matrices were computed under the K80 model (Kimura 2-parameter) (*62*), with gaps (insertions/deletions) excluded using the *pairwise.deletion* method. In parallel, a matrix of geographical distances between sampling localities was calculated from the coordinate data using the *distVincentyEllipsoid* function of the geosphere R package. The genetic and geographical distance matrices were both converted to long format and merged into a single data frame, so that each row contained a pairwise genetic distance, the corresponding geographical distance, and the associated OTU (and phylum). To ensure robust analysis, we first filtered the data to include only OTUs represented by at least four independent ASVs. Next, we further filtered these OTUs, called the informative OTUs, to retain only those in which at least one pair of sequences had a genetic distance of ≥0.01 and at least one pair had a geographical distance of ≥1 m. The ratio of the number of total OTUs and filtered informative OTUs per phylum can be downloaded from ratio_total_OTUs_informative_OTUs_by_phylum.csv in https://doi.org/10.6084/m9.figshare.29144645.v1 and the ID of the informative OTUs in the file informative_OTUs.txt of each phylum. This two-step filtering removed OTUs with very few sequences or with negligible variation, which could otherwise introduce noise. Metabarcoding studies that use general primers lack targeted taxonomic resolution and often retrieve fragmented sequences or sequences from multiple species, potentially biasing interpretations (*23*). Filtering out OTUs with uniformly low molecular divergence could mitigate these issues. While this approach might also exclude some genuine low genetic diversity cases (for example, populations that have undergone bottlenecks or are in decline), it primarily reduces spurious OTUs arising from methodological biases, improving the reliability of biogeographic pattern interpretations.

The results of each analyses of each informative OTUs were compiled into the Supplementary Data S3 and https://doi.org/10.6084/m9.figshare.29144645.v1. In this file, each row corresponds to an informative OTU and each column to the results of the analysis or metrics, such as Phylum (the taxonomic phylum of that OTU), OTU (the OTU identifier, distinct within each phylum), Max_Distance (the maximum geographical distance, in meters, between any two localities), Mean_Distance (the mean geographical distance among all locality pairs), Std_Dev_Distance (SD of geographical distances among localities), and Count (number of pairwise ASV comparisons, genetic/geographical distance pairs).

#### Within-OTU diversity and Tajima’s D

To quantify genetic diversity within each informative OTU, we calculated the nucleotide diversity (π). This was done using the previously generated alignments per phylum: the *nuc.div* function (*63*) was applied with *pairwise.deletion* = TRUE to compute π for each OTU using the script 9_calculate_nucleotide_diversity_by_otu.R. The resulting values were recorded in the Nucleotide_Diversity_Pi column of informative_OTUs_results.csv. We also calculated Tajima’s *D* for each informative OTU to investigate potential signals of selection or demographic history (e.g., recent expansion and bottleneck) using the script 10_calculate_tajimasD_by_otu.R. The *tajima.test* function (*63*) was applied to the alignment data for each informative OTU. In theory, a negative Tajima’s *D* indicates an excess of rare variants consistent with recent population expansion or purifying selection, whereas a positive Tajima’s *D* suggests a deficiency of rare variants, which may indicate a population bottleneck or balancing selection (*64*). Given our filtering criteria (which ensured multiple sequences per OTU and some level of genetic divergence), we expected Tajima’s *D* values to trend negative. Each OTU’s Tajima’s *D* value was recorded in a Tajima_s_D column, with its associated *P* values recorded in P_Value_Normal (normal approximation) and P_Value_Beta (β distribution approximation) columns.

#### Read count filtering impact

It is common in metabarcoding studies to discard ASVs with very low read counts (below a certain threshold) to avoid spurious ASVs (*65*). To evaluate how this filtering might affect the recovery of low-abundance haplotypes and OTUs, we characterized the occurrence of rare haplotypes within each OTU. First, for each informative OTU, the total number of ASVs and the total number of reads were recorded in the Num_Sequences and Total_Reads columns, respectively, in the informative_OTUs_results.csv file. In addition, separate columns captured the number of reads contributed by each ocean named <ocean>_reads. We then evaluate the potential loss of data and in reads counts filtering by counting the number of haplotypes with fewer than 10 reads for each informative OTU, recorded in the haplotypes_lt10 column. We also determined how many of these rare haplotypes were supported by at least two independent ASVs (each with <10 reads); this number was recorded in haplotypes_recovered_multiple column. By requiring multiple independent ASVs, we can identify rare haplotypes that are consistently observed, thereby reducing the likelihood of counting sequencing artifacts as true haplotypes. We also assessed whether the low-read filter could inadvertently affect more abundant haplotypes. For each OTU, we counted the number of haplotypes with total reads of >10 that nevertheless contained at least one constituent ASV with <10 reads. This count was recorded in haplotypes_gt10_with_lt10_seq column. Last, to examine the biogeographic distribution of low-abundance ASVs, we counted how many oceans had a total read count of <10 for each OTU. This value (the number of distinct oceans in which the OTU is represented by fewer than 10 reads) was recorded in the global_lt10 column. Given that metabarcoding studies are typically constrained to specific regions/oceans, any potential “tag jumping” across oceans (a form of cross-sample contamination) can be ruled out at this stage. Overall, these metrics allowed us to understand the extent to which stringent read filtering might exclude genuine but low-frequency haplotypes and the veracity of ASVs with low number of reads.

### Geographic and genetic distances

Linear regression and nonlinear analyses were conducted for each informative OTU to evaluate the relationship, strength, and statistical significance of the correlation between genetic and geographic distances using the R scripts 5_calculate_linear_models_by_otu.R and 6_calculate_nls_models_by_otu.R, respectively. The parameters of the linear model (lm) were calculated for each informative OTU using the dplyr R package, and the results were saved in the informative_OTUs_results.csv file, with the columns: Intercept_lm (intercept), Slope_lm (slope), R2_lm (coefficient of determination), P_value _lm (*P* value of the slope), AIC_lm (Akaike information criterion), and BIC_lm (Bayesian information criterion). In addition, a nonlinear regression analysis (nls) was performed using the model: “Genetic Distance” = *a* X log(“Geographical Distance” + 1) + *b*. Parameters *a* and *b* were estimated from predefined initial values. The output included the a_nls (slope parameter), b_nls (vertical offset parameter), RSS_nls (residual sum of squares), R2_nls (coefficient of determination), AIC_nls (Akaike information criterion), and BIC_nls (Bayesian information criterion). To interpret the differences in AIC and BIC between these two models, categories were established on the basis of the magnitude of the differences (Δ): (i) no evidence to prefer either model (|Δ| ≤ 2), indicating similar fit; (ii) moderate evidence (2 < |Δ| ≤ 7), indicating moderate support for one model; and (iii) strong evidence (|Δ| > 7), indicating strong support for one model (Supplementary Data S2). On the basis of these results, we continued with the nonlinear models for the graphical representations, as it better explained the genetic and molecular distance correlations per phylum.

Next, the linear and nonlinear models were compared with null models to assess whether the relationship between genetic and geographic distances was consistent with a stochastic pattern. The null models were generated by simulating random distributions of genetic and geographic distances using the Python script 7_calculate_null_models_by_otu.py, fitting linear models with the scikit-learn library and optimizing nonlinear models with SciPy’s curve_fit function. For each OTU, 1000 simulations were performed. In each simulation, the number of data points equaled the number of pairwise comparisons (count column in informative_OTUs_results.csv file) for that OTU. Geographical distances were sampled uniformly between 0 and the maximum recorded distance (max_distance column), and genetic distances were sampled uniformly between 0 and 0.03 (the specified OTU cutoff). From these simulations, the mean, median, and SD of *R*^2^, AIC and BIC were calculated for comparison with the observed models. Results were saved in informative_OTUs_results.csv file with the following columns: Mean_R2_lm_null, Median_R2_lm_null, Std_R2_lm_null, Mean_AIC_lm_null, Median_AIC_lm_null, Std_AIC_lm_null, Mean_BIC_lm_null, Median_BIC_lm_null, and Std_BIC_lm_null; and for nonlinear models: Mean_R2_nls_null, Median_R2_nls_null, Std_R2_nls_null, Mean_AIC_nls_null, Median_AIC_nls_null, Std_AIC_nls_null, Mean_BIC_nls_null, Median_BIC_nls_null, Std_BIC_nls_null, Mean_a_nls_null, Median_a_nls_null, Std_a_nls_null, Mean_b_nls_null, Median_b_nls_null, and Std_b_nls_null. *P* values were calculated to determine whether the observed relationships differed significantly from random expectations and were saved in the columns P_R2_lm_null (linear models) and P_R2_nls_null (nonlinear models).

To further examine whether a correlation exists between genetic and geographic distances and to assess its consistency across phyla, Mantel tests were performed using the R script 8_calculate_mantel_test_by_otu.R. For each OTU, genetic and geographic distance matrices were generated, and the Mantel test was conducted using the *mantel* function from the vegan package (version 2.6) (*66*) with 999 permutations; the results were recovered in the columns Mantel_R and P_Value_Mantel. However, the Mantel test can be problematic because of inherent dependencies in the data (*67*). In this context, the permutations used to calculate the *P* value may not adequately reflect the expected null distribution under true independence; therefore, this results should be interpreted with caution. After completing these analyses, only OTUs with a Mantel test *P* < 0.05 were retained, and comparisons between phyla were conducted as described above.

### Biogeography based on haplotype networks

#### Haplotype network construction and diversity metrics

Specific haplotypes were first identified from the alignment for each informative OTU using the *haplotype* function (*63*). The number of haplotypes per informative OTU was then recorded in a column named num_haplotypes. Next, using the *haploNet* function (*63*), haplotype networks based on minimum spanning networks were constructed for each informative OTU and used in subsequent analyses. We then calculate the haplotype diversity (*H*_d_), branch diversity (*B*_d_), and their combined metric (*H*_bd_) (*33*) using R script 11_calculate_haplotype_diversity_by_otu.R. We saved the results in columns named Hd, Bd, and HBd, respectively, in the informative_OTUs_results.csv file. In addition, the maximum value of the squared haplotype frequencies per OTU was recorded in a column Efh2_Hd.

The above metrics treat each ASV equally, regardless of its abundance (i.e., they are based only on ASV presence/absence within each haplotype). To account for the relative abundance of each ASV (as a proxy for its prevalence in the sample), we incorporated the associated read counts. Specifically, we used two metrics: (a) the total reads count for each ASV and (b) a logarithmic transformation, [log(reads + 1)], using the R script 12_calculate_weighted_haplotype_diversity_by_otu.R. By weighting sequences according to their total read counts and a logarithmic transformation, haplotypes with higher abundance have a greater influence on the calculated diversity metrics. Therefore, if a sequence-based diversity metric exceeds its read-weighted counterpart, then this indicates that rarer haplotypes disproportionately influence the haplotypic diversity (i.e., diversity is overestimated in the unweighted case). This approach is particularly useful for communities with high variability in abundances, as it allows patterns of dominance or inequality to emerge. However, it may underestimate diversity in OTUs that contain many low-abundance haplotypes. The results of these abundance-weighted calculations were recorded in the following columns: Hd_reads and Hd_log_reads (haplotype diversity); Bd_reads and Bd_log_reads (branch diversity); Hbd_reads and Hbd_log_reads (combined haplotype and branch diversity); and Efh2_reads and Efh2_log_reads (maximum squared haplotype frequencies).

#### Oceanic genetic structure

After calculating haplotype diversity metrics, we evaluated genetic structuring between and within populations (each corresponding to a different ocean). Analysis of molecular variance (AMOVA) was performed for each OTU using R script 13_calculate_amova_by_otu.R. OTUs were selected for AMOVA on the basis of the following criteria: (i) presence in at least two different oceans, (ii) at least two populations, and (iii) at least two distinct haplotypes within those populations. We used the *popr.amova* function to perform the AMOVA. Variance components and sums of squares were calculated for between-population and within-population comparisons. The results were saved in the informative_OTUs_results.csv file, in columns named Between_samples_SumSq, Within_samples_SumSq, Total_SumSq, Between_samples_Sigma, Within_samples_Sigma, and Total_Sigma. These metrics were then compared across phyla as described above. Because the sequence length is relatively short (∼313 base pairs), the AMOVA results should be interpreted with caution.

To mitigate potential biases introduced by short sequences, we evaluated genetic structuring among oceans using the haplotype frequencies and distributions for each informative OTU. The haplotypic diversity metrics total diversity (*H*_t_), within-population diversity (*H*_s_), genetic differentiation coefficient (*F*_st_), and gene flow (*N*_m_) were calculated for each OTU using script 14_calculate_Hs_fst_nm_by_otu.R. These four metrics were stored in columns Ht, Hs, Fst, and Nm; and comparisons between phyla were conducted as described above.

A modified haplotype diversity metric (*PH*_d_) was created to infer the distribution of haplotypes among populations (here defined by different oceans) using script 15_calculate_PHd_and_global_ocean_dominance_by_otu.R. This metric incorporates the relative distribution of haplotypes across populations. By using weighted relative haplotype frequencies, *PH*_d_ quantifies the contribution of population-level differences to the genetic diversity of each OTU. The classical haplotype diversity formula was adapted to account for the distribution of haplotypes across populations. In this adaptation, the relative frequency of each haplotype is redefined as its frequency in a given population divided by its total frequency in the OTU. The modified *PH*_d_ is therefore calculated as

$${\mathrm{PH}}_{d}=1-\sum_{\text{population}\left(\text{oceans}\right)}{\left(\frac{\text{number of haplotypes in the population}}{\text{Total number of haplotypes in the}\;\text{OTU}}\right)}^{2}$$

The *PH*_d_ values were saved in a column named PHd. The relative haplotype frequencies for each ocean were stored in columns named <ocean>_sqfreq. These *PH*_d_ metrics were then compared across phyla using the same procedure as for the other diversity metrics. Furthermore, we compare *PH*_d_ with *H*_s_ metric to help identify whether the populations of an informative OTU are genetically structured or relatively homogeneous across oceans. If *PH*_d_ exceeds *H*_s_ (a positive difference), then this indicates that the populations have distinct genetic compositions and are partially isolated from one another, reflecting significant genetic structuring and no single population dominating the haplotype diversity. Conversely, if *PH*_d_ is less than *H*_s_ (a negative difference), then one or a few populations account for most of the global haplotypic diversity in the OTU, suggesting that these populations dominate the haplotypic diversity.

To assess the effect of aggregating taxa with contrasting life histories, we used Ochrophyta as a case study. We split all OTUs taxonomically annotated to Phaeophyceae (multicellular brown algae) from the remaining Ochrophyta lineages (predominantly unicellular; e.g., diatoms). For each subset, we recomputed the population-genetic metrics described above. The resulting summary statistics are provided in Supplementary Data S6 and https://doi.org/10.6084/m9.figshare.29144645.v1.

For each informative OTU, we identified the ocean with the highest haplotype relative contribution (the maximum sqfreq value) as a proxy for the putative population of source and vice versa for recent introduction populations. However, this analysis is considered a preliminary proxy to guide further investigation. To distinguish likely source populations from recent introductions, we calculated ratios of relative haplotype frequencies between oceans using script 20_introductions_vs_dominant_oceans_byOTU.R. Specifically, within each OTU, we used the ocean with the highest relative frequency as the reference (ratio = 1), and we computed for each other ocean the ratio of the reference frequency to that ocean’s frequency. We then examined the distribution of these ratios across all informative OTUs to detect shifts toward lower values. A notable change in the distribution occurred at a ratio of 0.25. Consequently, for each OTU, we classified any ocean with a ratio below 0.25 as a “recently introduced ocean/population,” since these oceans have very low relative haplotype frequency compared to the highest haplotypic frequency ocean. Furthermore, to identify a clear source ocean for each informative OTU, we applied additional filtering criteria. We selected OTUs in which exactly one ocean had a ratio of 1.0 (i.e., a single ocean had the maximum relative frequency) and excluded informative OTUs where multiple oceans shared this maximum frequency, since that would preclude a clear source. We also excluded informative OTUs in which any other ocean had a ratio exceeding 0.5 to avoid ambiguity. Although these thresholds are somewhat arbitrary, they provide a more restrictive criterion that allows a more reliable inference of the putative source ocean for the remaining informative OTUs. We create a pie chart graph of the proportion of dominant oceans per phylum (ancestral_oceans.pdf). Furthermore, we combine the number of OTUs dominant and introduced oceans in the dominant_introduced_oceans.csv file (in https://doi.org/10.6084/m9.figshare.29144645.v1).

To validate this proxy for source (dominant) and introduced ocean populations, we used phylum Chordata taxonomically annotated informative OTUs as a positive control. We included only those informative OTUs that had at least one ocean classified as “recently introduced.” For each selected informative OTU, the first ASV was compared to entries in the National Center for Biotechnology Information database using BLAST (*68*). We retained those OTUs for which the BLAST hit showed ≥97% sequence identity to a species in the database, ensuring that species-level identification was feasible. Taxonomic annotation results for these OTUs are recorded in introduced_chordata_otus.csv file (in https://doi.org/10.6084/m9.figshare.29144645.v1).

For each informative OTU, we inferred connectivity between different oceans from its haplotype network to evaluate genetic dispersal and exchange. This analysis was performed using the script 16_generate_ocean_connectivity_by_otu.R. In each haplotype network, nodes represent haplotypes, and edges represent connections between them. We annotated each edge with the ocean of origin for each haplotype in the pair. Only connections linking haplotypes from different oceans were retained; intraoceanic connections were discarded because they do not inform interocean connectivity. We then aggregated the data to count the number of connections between each pair of oceans. For each informative OTU, we recorded the total number of oceanic haplotypic connections in the column Total_Connections and the number of connections per ocean in columns named <ocean>_connections. Then, we classified oceanic pairs as “nearby” if they are geographically adjacent and “distant” if they are geographically separated using the script 17_calculate_ocean_conectivity.R; the number of connections was recovered in the columns nearby_oceans_connections and distant_oceans_connections. OTUs in which all haplotypes occur in the same ocean therefore have only nearby connectivity and no distant connectivity. We also characterized intrahemispheric and interhemispheric connectivity, focusing on connections between oceans in the Northern and Southern Hemispheres, recovered in the columns Inter-Hemisferica and Intra-Hemisferica.

For each phylum, we generated network graphs illustrating connections among oceans using the script 18_oceanic_network_by_phyla.py and 19_global_oceanic_network.py. In these graphs, each node represents an ocean, and each edge represents the total number of connections between two oceans, weighted by relative strength. We summed all connections across OTUs for each phylum and applied a log1p transformation (the natural logarithm of one plus this sum) to normalize the data. We then applied the Louvain community detection algorithm to identify clusters (communities) within each network, coloring nodes by their community membership. The networks were constructed using the networkx library, with edge weights reflecting connection strength the network per phylum were recovered as network_<phylum> .pdf and .jpg.

### Statistical test

For each variable generated per OTU, we examined whether there were significant differences among phyla. We first checked the distribution of each variable: The Shapiro-Wilk test indicated that the data were not normally distributed. Therefore, a nonparametric Kruskal-Wallis test (using the *kruskal.test* function in R) was used to test for differences among phyla. A significant Kruskal-Wallis *P* value was taken as evidence of variation among phyla for the given metric. In cases of significance, post hoc pairwise comparisons were performed using Dunn’s test (*dunnTest* function in R) with a Bonferroni adjustment for multiple testing (*69*). The results of the pairwise comparisons for each variable were saved in Supplementary Data S4 and https://doi.org/10.6084/m9.figshare.29144645.v1. The file contains the columns Z (*Z* statistic), P_unadj (uncorrected *P* value), and P_adj (adjusted *P* value) for every variable in the phyla pairwise comparison. Phyla with an insufficient number of informative OTUs, less than four, were excluded from these comparative analyses (Filasterea, Hemichordata, Nibbleridia, Prasinodermophyta, and Ustilaginomycotina). In addition, to infer differences between and within groups, phyla were grouped into three broad categories (Archaeplastida, Metazoa, and all other phyla) to compare the prevalence of significant differences between and within these groups (Supplementary Data S7 and https://doi.org/10.6084/m9.figshare.29144645.v1).

Last, we analyzed correlations among the different calculated variables for each informative OTU. We computed correlation matrices and associated *P* values using Pearson’s correlation coefficient, generating a table listing only those correlations that were statistically significant (*P* < 0.05) (Supplementary Data S5 and https://doi.org/10.6084/m9.figshare.29144645.v1).
