# Supplementary material for: Marine population-genetic inferences reveal stronger oceanic structure in protists than Archaeplastida (plants) and Metazoa (animals)
Source: Sci Adv. 2026 Jun 10;12(24):eadz7158. doi: 10.1126/sciadv.adz7158 (PMC13251859; doi:10.1126/sciadv.adz7158)
Supplement: Supplementary file 1 — Figs. S1 to S9 Legends for data S1 to S7 [file sciadv.adz7158_sm.pdf]

Science Advances
AAAS

Supplementary Materials for

Marine population-genetic inferences reveal stronger oceanic structure in protists than Archaeplastida (plants) and Metazoa (animals)

Rubén González-Miguéns *et al.*

Corresponding author: Rubén González-Miguéns, ruben.miguens@ibe.upf-csic.es;
Iñaki Ruiz-Trillo, inaki.ruiz@ibe.upf-csic.es

Sci. Adv. **12**, eadz7158 (2026)
DOI: 10.1126/sciadv.adz7158

The PDF file includes:

Figs. S1 to S9
Legends for data S1 to S7

Other Supplementary Material for this manuscript includes the following:

Data S1 to S7

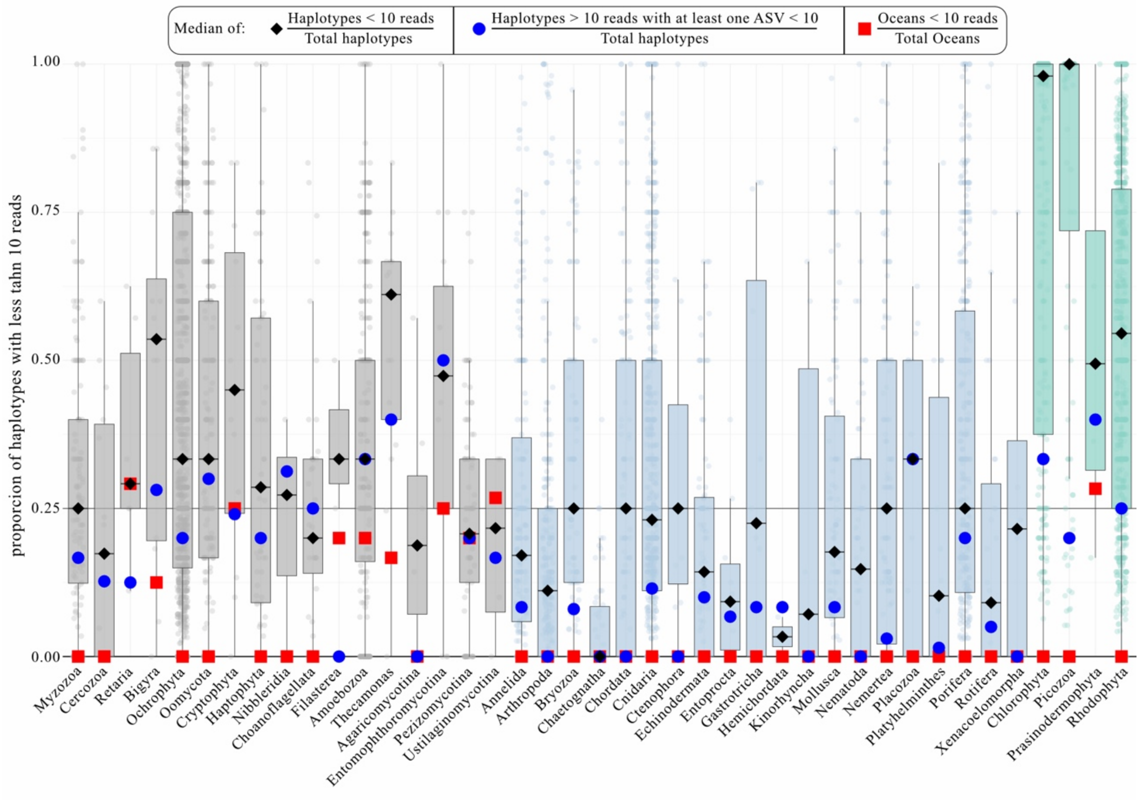

Fig. S1. Rare haplotypes (< 10 reads). Boxplots (by phylum) show the distribution, across OTUs, of the proportion of haplotypes with fewer than 10 total reads. Black diamonds indicate the median, and box colors denote higher-level taxonomic groups. Blue circles mark the median proportion of haplotypes with >10 total reads that include at least one constituent ASV with <10 reads. Red squares mark the median proportion of oceanic regions with <10 reads for the corresponding OTUs.

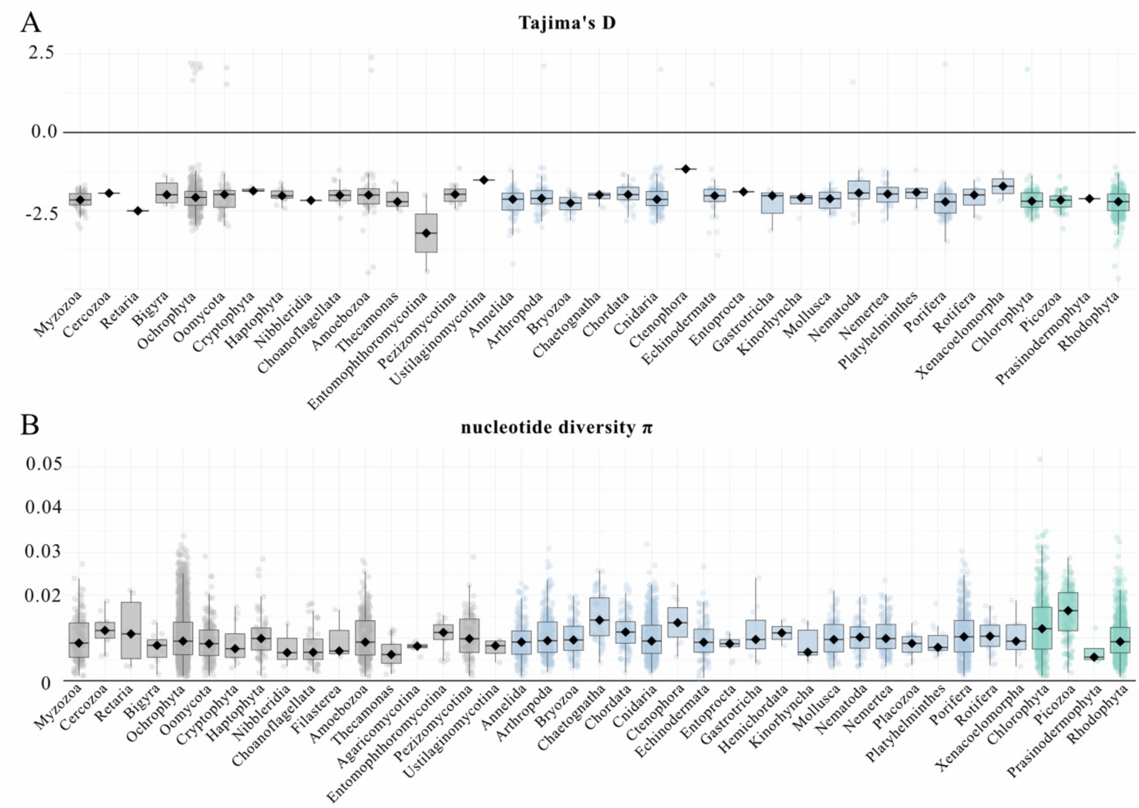

Fig. S2. Nucleotide diversity metrics. (A) Boxplot representing the values of Tajima's D for OTUs grouped by phylum (significant tests only; $p < 0.05$). (B) Boxplot of nucleotide diversity (π) values for OTUs grouped by phyla.

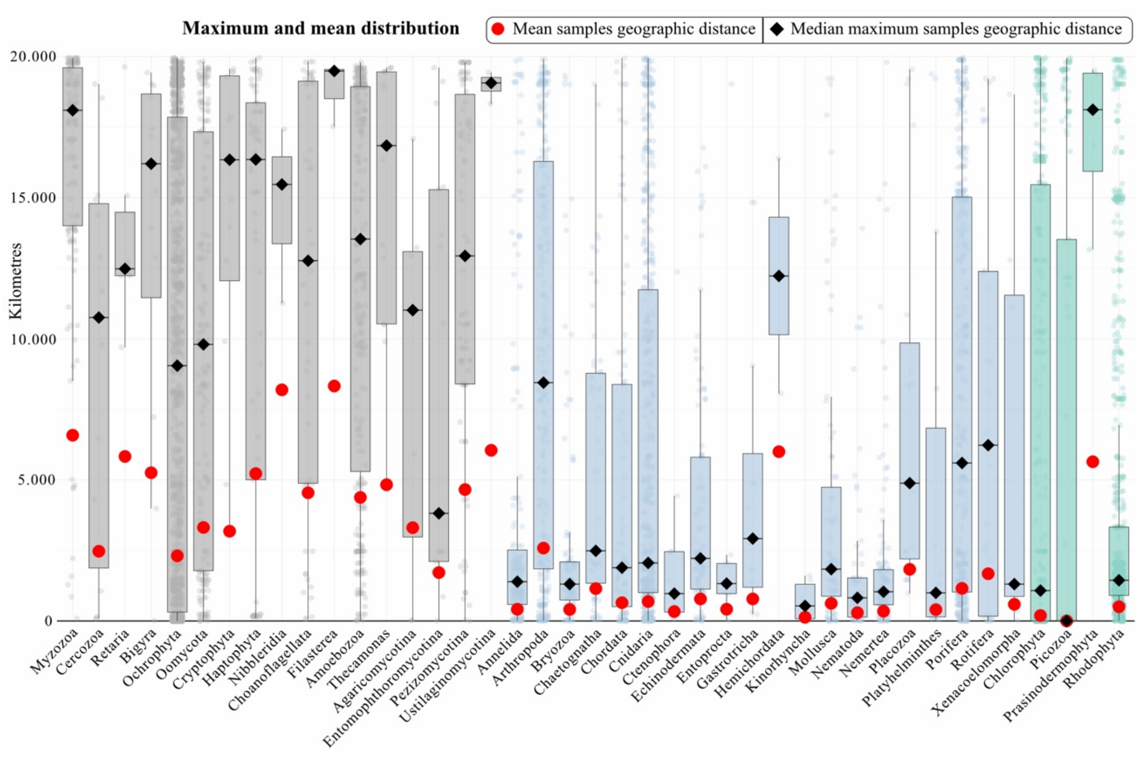

Fig. S3. Geographic distances. The boxplots (grouped by phylum) show, for each informative OTU, the maximum pairwise geographic distance between sampling localities. Red circles indicate the median of the mean geographic distance across all locality pairs within OTUs.

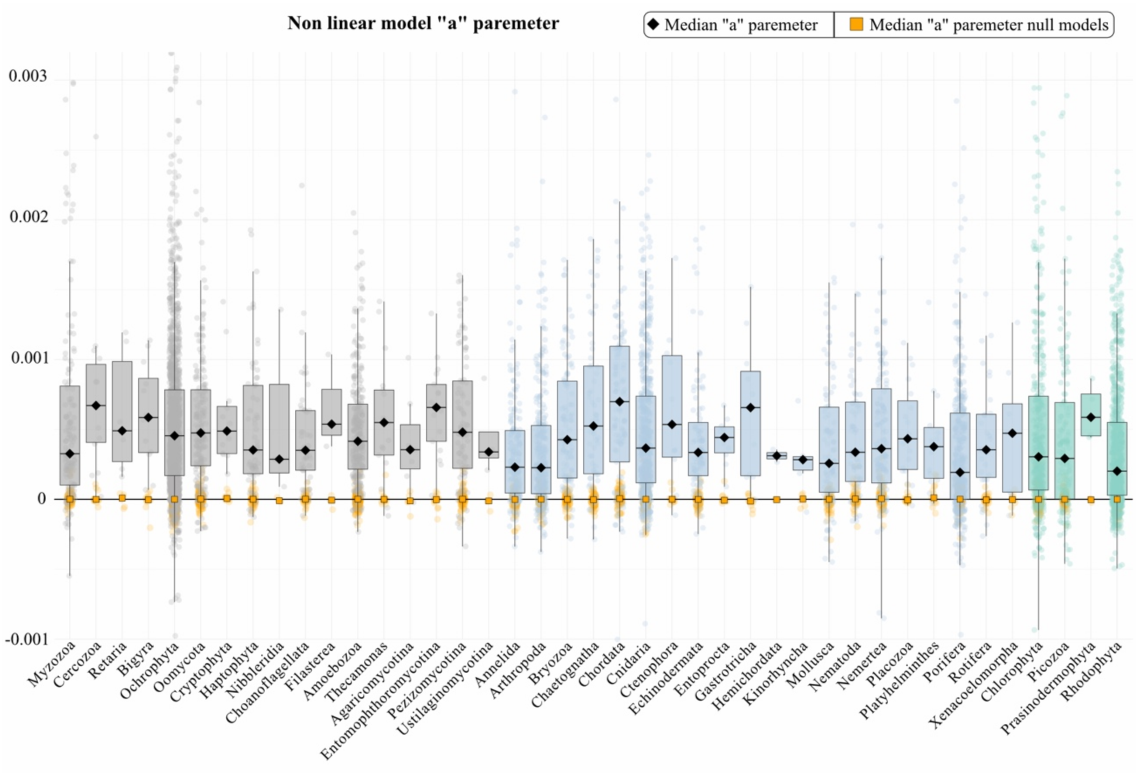

Fig. S4. Parameter "a" of the nonlinear (exponential) model. Boxplots show estimates of parameter "a" from the nonlinear (exponential) fits relating genetic to geographic distance for each informative OTU (grouped by phylum). Orange points denote values from null models generated for each phylum; orange squares mark their medians.

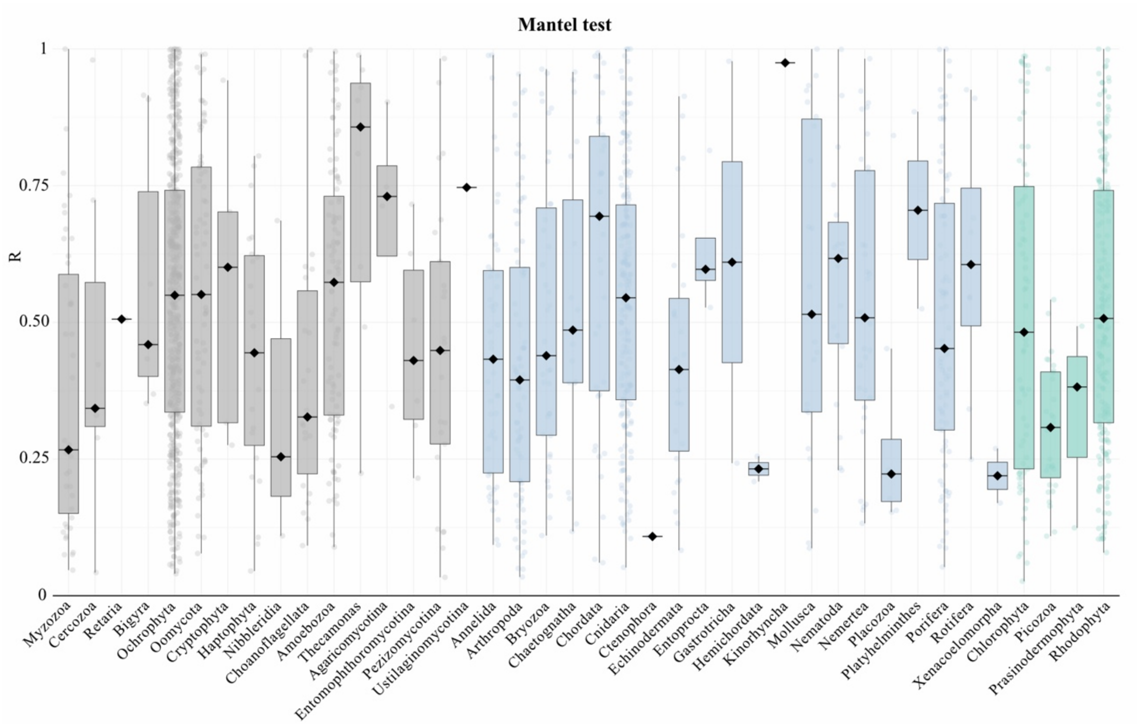

Fig. S5. Significant Mantel test. Boxplots show Mantel correlation coefficients (r) for informative OTUs with $p < 0.05$.

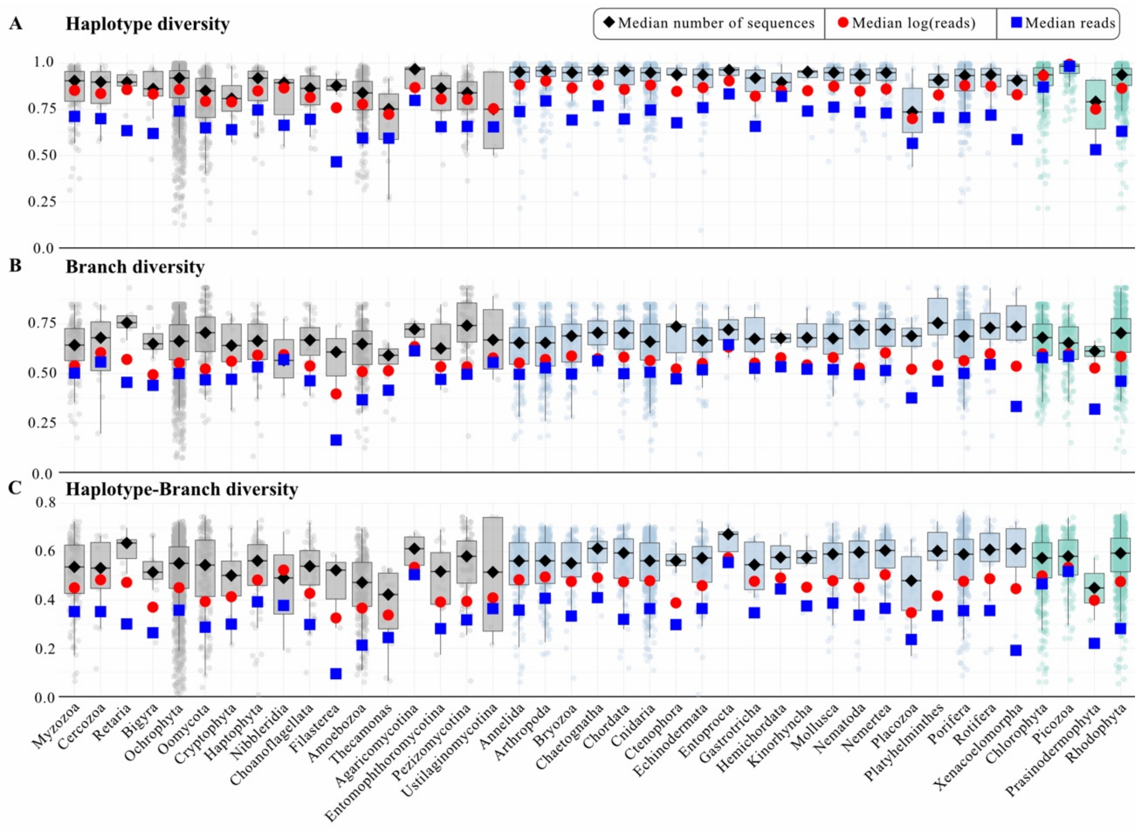

Fig. S6. Haplotypic diversity metrics. (A) Boxplot representing haplotype diversity values, (B) branch diversity, and (C) haplotype-branch diversity, all based on the number of sequences. In all three cases, the median considering the number of sequences is represented by diamonds, the median based on total reads is represented by squares, and the median using log(reads) is represented by circles.

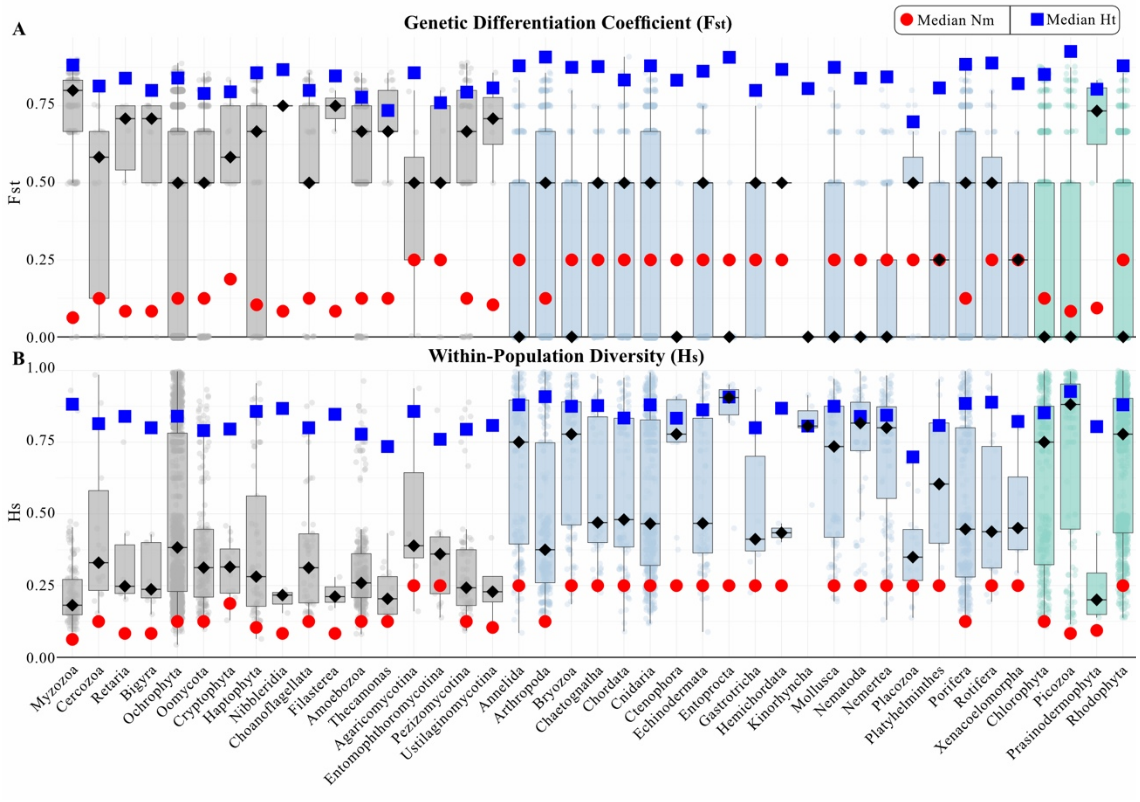

Fig. S7. Population structure. (A) Boxplot representing the F_{st} values, and (B) boxplot of H_s values per informative OTU grouped by phyla. Circles represent the median of N_m values, and squares represent the medians for H_t values.

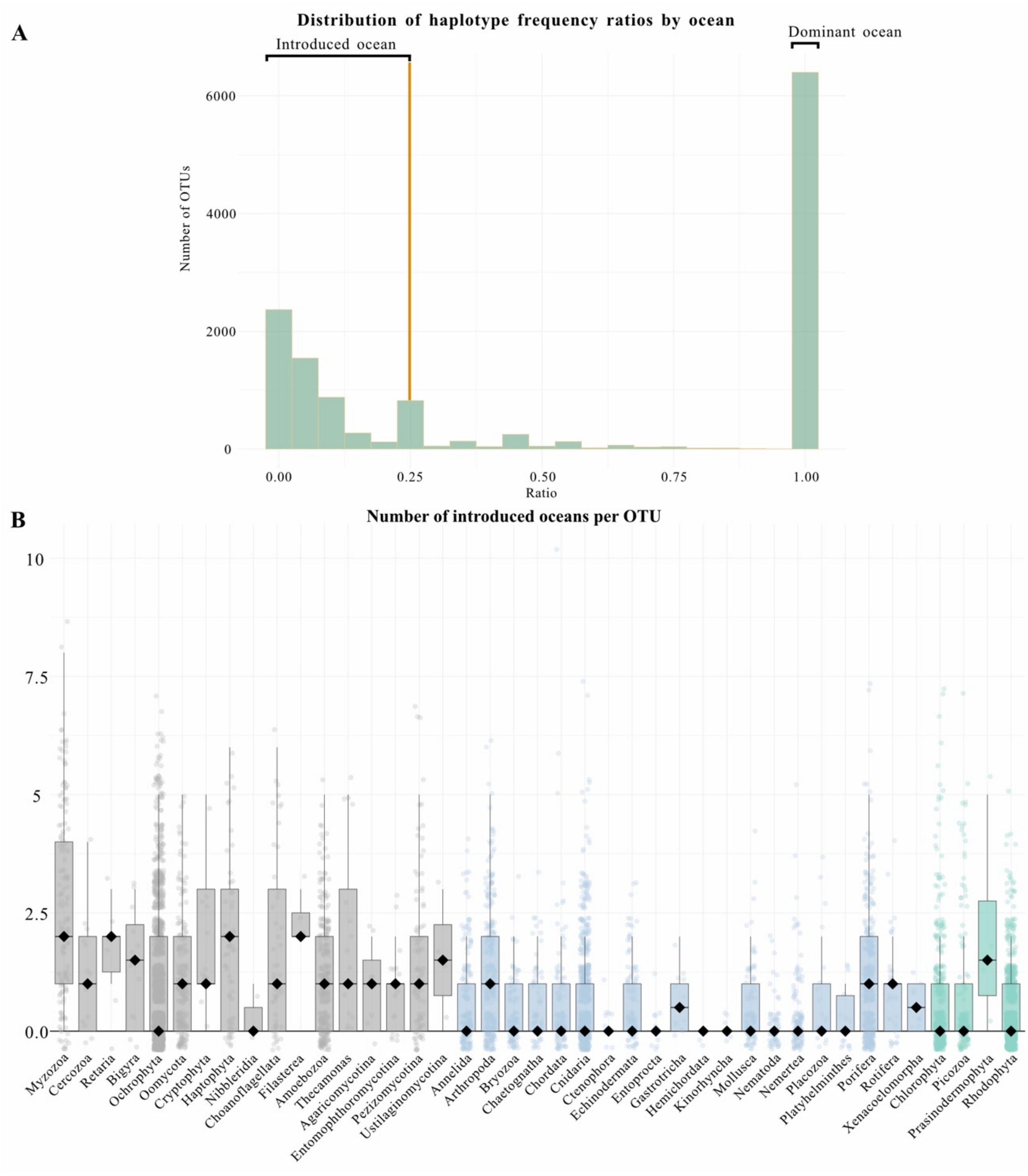

Fig. S8. Characterization dominant and introduced ocean per OTU. (A) Distribution of haplotype relative frequency ratios between oceans of all informative OTUs. (B) number of introduced oceans per informative OTU grouped by phyla.

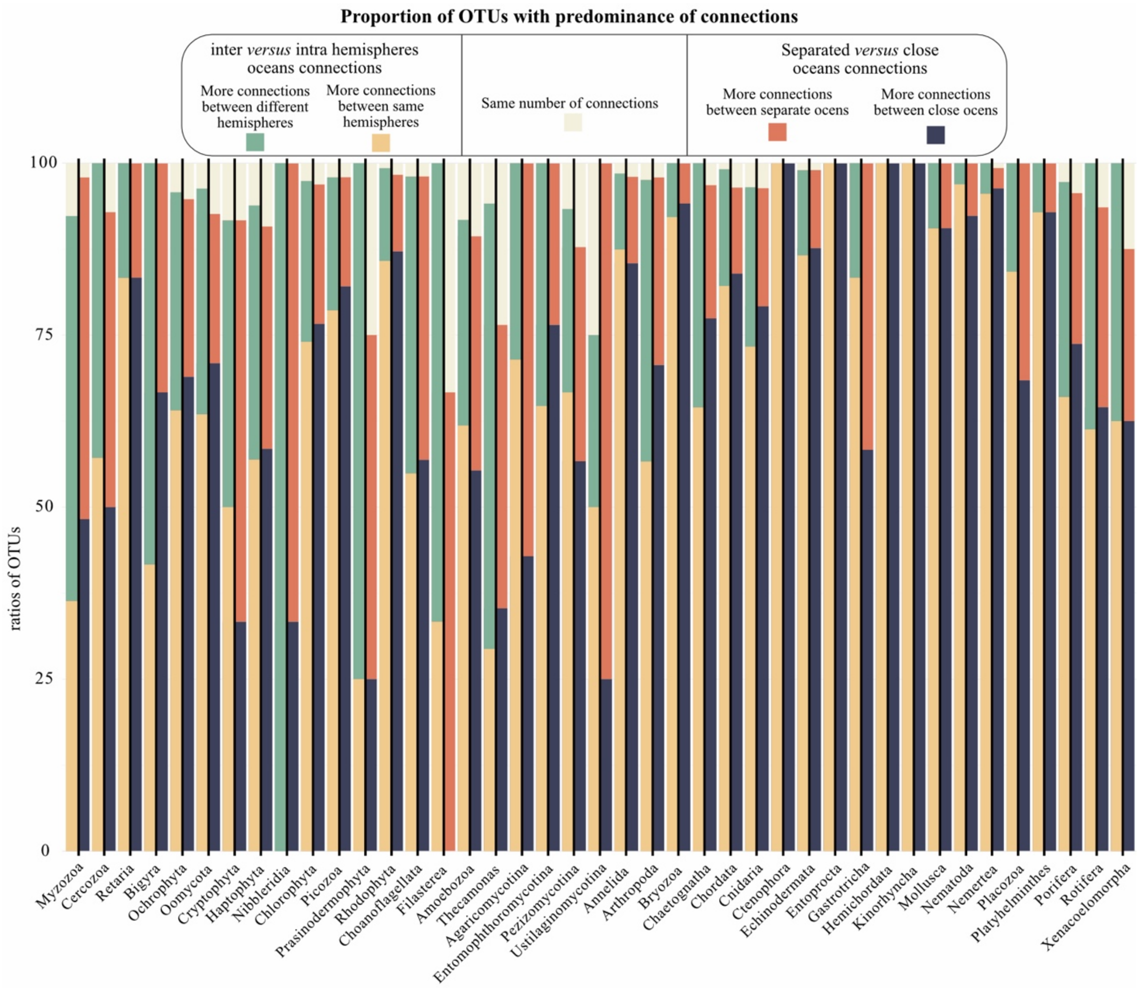

Fig. S9. Ratios of haplotype oceanic connections. Bar plot representing the composition of the ratios for the different types of ocean connections, based on the haplotypic networks, for each phylum. The left side of the bar plot illustrates the proportion of connections between inter- versus intra-hemispheric oceans, while the right side shows the proportion of connections between separated versus adjacent oceans.

Other Supplementary Materials for this manuscript include the following:

Data S1 to S7.

Data S1.
eKOI_metabarcoding_database_taxonomic_annotations.xlsx: Taxonomic annotation of each ASV based on the eKOI taxonomic reference database.

Data S2.
models_comparison_summary.csv: Summary of model comparisons for each phylum.

Data S3.
informative_OTUs_results.csv: Results of all analyses performed for each informative OTU.

Data S4.
significant_comparisons_Dunn_test.csv: Results of pairwise Dunn's tests across phyla for each variable analyzed, including Z statistics, unadjusted p-values (P_unadj), and Bonferroni-adjusted p-values (P_adj).

Data S5.
significant_correlations.csv: Significant Pearson's correlations among variables for the complete dataset of informative OTUs. Only statistically significant correlations are reported.

Data S6.
Phaeophyceae.csv: Population-genetic metrics for OTUs assigned to Ochrophyta, separating those taxonomically identified as Phaeophyceae from the remaining ochrophyte OTUs.

Data S7.
significant_group_comparisons_Dunn_test.csv: Summary of the number of statistically significant pairwise comparisons between phyla (Dunn's test with Bonferroni correction), relative to the total possible comparisons. Also includes the number of significant comparisons between the three broader groups (Archaeplastida, Metazoa, and all other phyla).
